# Role of eculizumab in a pediatric refractory gemcitabine-induced thrombotic microangiopathy: a case report

**DOI:** 10.1186/s13256-017-1373-5

**Published:** 2017-07-27

**Authors:** Ludovica Facchini, Maurizio Lucchesi, Alessia Stival, Rosa Maria Roperto, Francesca Melosi, Marco Materassi, Silvia Farina, Veronica Tintori, Maurizio de Martino, Iacopo Sardi

**Affiliations:** 1 Neuro-Oncology Unit, Department of Pediatric Oncology, Meyer Children’s Hospital, Viale G. Pieraccini 24, 50139 Florence, Italy; 2 Nephrology Unit, Meyer Children’s Hospital, University of Florence, Florence, Italy; 3 Intensive Care Unit, Meyer Children’s Hospital, Florence, Italy; 4 Transplantation Unit, Department of Pediatric Oncology, Meyer Children’s Hospital, Florence, Italy; 5 Department of Health Sciences, University of Florence, Meyer Children’s Hospital, Florence, Italy

**Keywords:** Microangiopathy, Eculizumab, Gemcitabine, Medulloblastoma, Radiotherapy, Brain tumors, Case report

## Abstract

**Background:**

The incidence of gemcitabine-induced hemolytic uremic syndrome has already been described in adults. Several approaches have been employed in the treatment of gemcitabine-induced hemolytic uremic syndrome with different outcomes. One of the most promising agents is eculizumab, which is a monoclonal antibody directed against C5 complement protein.

**Case presentation:**

We reported the case of a 3-year-old white boy with medulloblastoma who underwent high-dose chemotherapy and craniospinal irradiation. Afterwards he started maintenance chemotherapy with gemcitabine and oxaliplatin. After five courses he presented a progressive clinical worsening, which resulted in a systemic thrombotic microangiopathy. Initially he was treated with rituximab without clinical improvement. Therefore he started therapy with repeated cycles of eculizumab. After seven infusions he showed a gradual improvement and finally a complete remission of gemcitabine-induced hemolytic uremic syndrome.

**Conclusions:**

Eculizumab prevents serious complement-mediated vascular damage for chemotherapy-induced thrombotic microangiopathy in pediatric cases.

## Background

Eculizumab is a monoclonal antibody directed against C5 complement protein preventing formation of C5a and C5b-9 membrane-attack complex [1]. It shows a low toxicity profile and seems to be well tolerated in patients with paroxysmal nocturnal hemoglobinuria and atypical hemolytic uremic syndrome (aHUS). Eculizumab has been approved for use in children with aHUS, and a pediatric weight-based dosing schedule has been suggested [2]. The role of eculizumab in children with hematopoietic stem cell transplantation-associated thrombotic microangiopathy (HSCT-TMA) is still uncertain, but a recent study reported encouraging results and suggested the possible therapeutic dosage of the drug [3].

A meta-analysis on thrombotic microangiopathy in adults reported an incidence of 0.25 to 0.4%, an onset after a cumulative dose of 2000 to 48,000 mg/m^2^ and after 5 to 8 months of treatment, an evidence of renal failure in 34 up to 69% of the patients, and a severe prognosis with a mortality up to 60% [1]. GiHUS may not present the typical signs of hemolytic uremic syndrome (HUS); therefore, the diagnosis can often be delayed. Creatinine increase and uncontrolled hypertension or worsening of a pre-existing hypertension may be the only signs for early detection of HUS [4, 5]. Unfortunately, there is not a reliable approach for GiHUS even if an immediate discontinuation of gemcitabine is the common initial step [6]. Several therapies, including high-dose corticosteroids, plasmapheresis, antihypertensive drugs, and rituximab, have been employed in the treatment of GiHUS with different results [4, 7, 8]. Al Ustwani *et al.* reported the use of eculizumab in GiHUS after failure in clinical improvement with other therapies [4]. The outcome was a resolution of the microangiopathic hemolysis and thrombocytopenia. Despite significant improvement in renal function, the creatinine level remained above its average values. Thus, eculizumab appeared to be a well-tolerated, safe and effective treatment for GiHUS [4].

We report a case of GiHUS arising in a boy with an overtreated medulloblastoma, highlighting the substantial role of eculizumab in the pediatric population.

## Case presentation

A 3-year-old white boy with a history of headache, abdominal pain, and walking abnormalities of 6 months’ duration was admitted to our hospital with worsening ataxia, intense headache, and irritability. A brain computed tomography (CT) scan revealed a mass in his posterior fossa causing hydrocephalus; therefore, he underwent an endoscopic third ventriculocisternostomy. Two days later a magnetic resonance imaging (MRI) scan confirmed the presence of a lesion in his posterior fossa associated to spinal nodules (C4, C6, C7). Therefore he underwent a gross total resection with good motor function recovery. The histopathological diagnosis was classic medulloblastoma. An analysis of cerebrospinal fluid (CSF) showed positive cytology for neoplastic cells. He started a chemotherapy program with two courses of high-dose regimen and autologous stem cell rescue after an induction phase, according to the Italian protocol for infants with high-grade central nervous system (CNS) tumors [9]. At the end of treatment a MRI scan showed complete remission and the CSF cytology was negative for neoplastic cells. The child began the follow-up when after only 4 months a MRI scan showed contrast enhancement at cerebellum and spinal cord (C1 to C3, D11), which was consistent with disease progression. Therefore, he underwent a second-line therapy with weekly vinorelbine 30 mg/m^2^ and craniospinal hyperfractionated accelerated radiotherapy with a total dose of 31.2 Gy on the craniospinal axis (bi-fractionated, 1.3 Gy/fraction), a boost dose of 59.7 Gy (bi-fractionated, 1.5 Gy/fraction) on his posterior fossa, and a boost dose of 9 Gy on cervical C1 to C3 and dorsal D11 levels (1.4 Gy/fraction) [10]. The MRI evaluation after radiotherapy showed a good response and he started maintenance chemotherapy with gemcitabine-oxaliplatin cycles (750 mg/m^2^ and 75 mg/m^2^ respectively, reducing the doses to 75% of the total) every 21 days for a total of five courses, which lasted 12 weeks.

Five months after the beginning of gemcitabine therapy, his overall clinical condition worsened. A physical examination revealed only pulmonary clinical signs, specifically crackles localized in the middle field of his right lung. A chest X-ray showed a slight accentuation of bilateral perihilar peribronchovascular interstitium. Concomitantly he was transfused for the detection of acute anemia and thrombocytopenia.

**Fig. 1:**
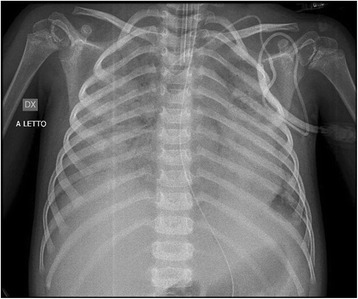
A chest X-ray scan showing bilateral diffuse acute distress respiratory syndrome at Intensive Care Unit admission

**Fig. 2:**
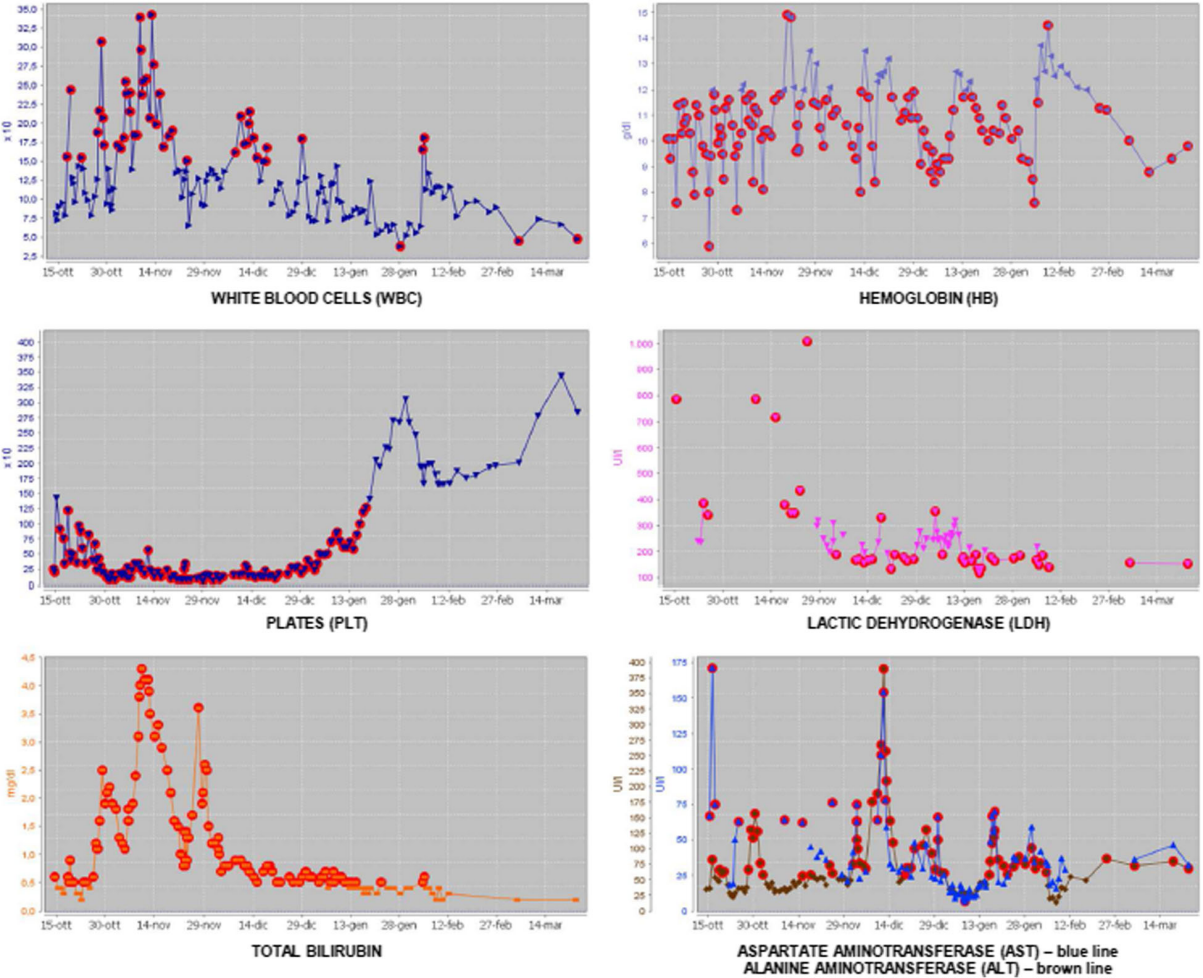
Blood tests of thrombotic microangiopathy (*red circles* in graphs mark values out of range)

**Fig. 3:**
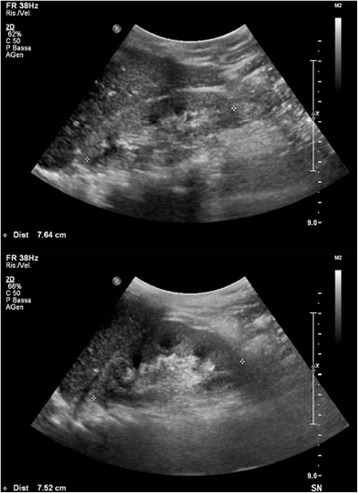
An echo scan of the kidneys showing a diffuse parenchyma hyperechogenicity, consistent with bilateral nephropathy

After platelet transfusion a sudden dyspnea appeared and, with the suspicion of an allergic reaction, chlorpheniramine maleate was administered without clinical improvement. A second chest X-ray showed a diffuse interstitiopathy and an initial right costophrenic effusion (Fig. 1). Empirical antibiotic therapy with ciprofloxacin and teicoplanin was started. Blood cultures were negative. Captopril and furosemide were administered because of the appearance of high blood pressure and initial acute renal failure. Anemia and thrombocytopenia persisted with a worsening of his pulmonary and renal impairment at each transfusion. Therefore, hypothesizing a thrombotic microangiopathy (TMA), he was no longer transfused. After an event of acute respiratory distress syndrome (ARDS), he was transferred to the Intensive Care Unit of our hospital. He was subjected to renal ultra-filtration and high-flow oxygen and therapy with amine in combination with broad spectrum antibiotics and antifungals. Serology results for viruses, bacteria, and fungi were negative. Several tests were carried out on blood samples and bronchoalveolar lavage. Finally, polymerase chain reaction for cytomegalovirus, Epstein–Barr virus, respiratory syncytial virus, adenovirus, *Mycoplasma pneumoniae*, *Klebsiella*, pneumococcus, *Staphylococcus*, and pneumocystosis were also negative. The involvement of lung (ARDS) and kidney (renal failure treated with dialysis), the hypertension associated with hemolytic anemia and thrombocytopenia, and the undetectable haptoglobin made us suspect an antibody-mediated microangiopathy. His disintegrin and metalloproteinase with a thrombospondin type 1 motif, member 13 (ADAMTS 13), activity was normal. The dosage of the complement proteins C3 and C4 was within normal limits. Thus, he was treated with defibrotide, four cycles of rituximab with a transient response, immunoglobulins, and plasma exchange. Considering his persistent poor clinical condition, with suspicion of a complement-mediated microangiopathy, he started a therapy with eculizumab at a dosage of 600 mg every 3 weeks for a total of seven infusions. He was previously vaccinated for meningococcus and started antibiotic prophylaxis. His neurological status considerably improved within 48 hours after the first infusion, then we observed an improvement in his general clinical condition and of the laboratory disease activity markers; he was rapidly dismissed from the Intensive Care Unit (Figs. 2 and 3). He continued the follow-up showing a gradual improvement in his clinical condition: he no longer showed any respiratory symptoms and he recovered his kidney function progressively, reducing the frequency of dialysis sessions until he did not need it anymore. On the neurological side, his neurocognitive functioning gradually improved; his interaction and speech improved. In fact we observed an increase in platelets and red cells count and sufficiently stable levels.

Unfortunately, after seven infusions, there were no improvements, so the therapy with eculizumab was suspended. After 1 month he showed respiratory difficulties with almost daily episodes of apnea. MRI scans evidenced radionecrosis areas on the medulla, which disappeared after a month of corticosteroid therapy. Subsequently, he presented two episodes of pneumonia in bone marrow aplasia. Five months after the end of the previous treatment with eculizumab, considering his respiratory difficulties associated with new episodes of apnea, we decided to re-administer therapy with eculizumab suspecting a microangiopathy reactivation. In fact blood test results showed a worsening anemia and thrombocytopenia, which confirmed our suspicion.

Despite two cycles of eculizumab carried out in 1 month, unfortunately he died from a sudden cardiovascular instability, with persistent respiratory distress, peripheral edema, and pericardial effusion.

## Discussion

We described a boy with a rare heavily treated medulloblastoma who developed a TMA as a complication of chemotherapy with gemcitabine (cumulative dose of 3750 mg/m^2^). The early clinical presentation appeared 5 months after the beginning of gemcitabine treatment and was characterized by anemia, thrombocytopenia, and hypertension. Afterwards he developed an acute respiratory syndrome associated with transfusion of platelets and red cells. Despite treatment with dexamethasone, rituximab, and plasmapheresis we did not observe any clinical improvement. He had a good recovery after eculizumab with a dosage of 600 mg (as suggested for aHUS in patients >40 kg of weight) confirming the suspicion of a complement-mediated microangiopathy. Unfortunately, we were not able to study eculizumab levels and the complement activity, which could have helped us to assess whether our patient needed a dosage adjustment. As, according to Jodele *et al.*, achieving therapeutic drug levels or complement blockade seemed to be related to therapeutic success; furthermore, they suggested that children with HSCT-TMA require higher eculizumab dosing or more frequent drug administrations, to reach and maintain therapeutic eculizumab levels, compared with the dosing regimen currently recommended for children with aHUS [3]. Our patient showed a rapid response after the first infusion and a gradual increase of renal function after the following doses and we were able to stop eculizumab infusion, since we achieved the remission of the TMA. We monitored the disease, and started another eculizumab infusion when we suspected a recurrence, which we observed 5 months after remission of TMA and last infusion of eculizumab.

Many adverse events were reported for use of gemcitabine; bone marrow depression is the dose-limiting toxicity. In children were reported cardiovascular effects (edema related to heart failure), neurologic effects (drowsiness, paresthesia), gastrointestinal and genitourinary symptoms (diarrhea, nausea and vomiting, stomatitis, hepatic toxicity ematuria, proteinuria, renal failure), respiratory symptoms (bronchospasm, dyspnea, flu-like symptoms) and fever. TMA was reported as a rare but dangerous adverse event [11].﻿﻿ The incidence of GiHUS has been reported to be however low: between 0.015 and 2.2% [1, 4, 5]. Literature data only concern adult patients treated with gemcitabine for different types of cancer such as non-small cell lung, ovarian, metastatic breast, gastric, and pancreatic cancers [1, 4–8, 12]. At present no specific risk factors in developing GiHUS have been identified due to the small number of cases and to the heterogeneity and complexity of the patients with chronic and serious disease. The median time between initiation of gemcitabine chemotherapy and onset of GiHUS was 7.4 months [5]. Our pediatric patient developed GiHUS 5 months after the beginning of gemcitabine treatment. No clear dose–response relationship between gemcitabine treatment and development of GiHUS was described: the median cumulative dose is 20 g/m^2^ with a broad range from 2.45 to 48 g/m^2^ [5].

Intriguingly, the TMA developed after treatment with gemcitabine could be associated with the two courses of high-dose thiotepa and autologous stem cell rescue that our patient underwent 12 months before. In this respect, some studies also recently proposed repeated eculizumab infusions for TMA associated with hematopoietic stem cell transplantation [1–3, 13].

Over the years, different types of treatment with variable responses have been proposed. Several groups mainly considered immunosuppressive therapies with corticosteroids, and more recently with rituximab [12, 14–16]. Although these therapies seem to be safe and quite effective, HUS remains a highly fatal disease with a mortality rate ranged as high as 60 to 70% in some reports [16–18].

A better knowledge about the role of complements in chemotherapy-induced TMA could help as a rationale for the use of eculizumab. TMA affects both renal and extrarenal microvasculature including involvement of the lungs, liver, whole gastrointestinal tract, central nervous system, cardiovascular system, and other systems and organs. It is noteworthy that the excess in complement activation is related to severe thrombophilia. Complement activation could aggravate in the setting of complement regulatory defects and further contribute to thrombotic occlusions in the microvasculature and even macrovasculature [19, 20]. Therefore the efficacy of eculizumab in some cases with GiHUS supports the prominence of complement-mediated mechanisms [4, 7, 8].

Although the usage of eculizumab in infancy is limited, a recent report by Yüksel and coworkers showed that this agent is a safe and effective drug in pediatric patients [21].

## Conclusions

In conclusion, in accordance with reports on cases of adult patients, we obtained a sudden response to eculizumab therapy in a pediatric case after the diagnosis of complement-mediated microangiopathy [22].

Therefore, we wish to stimulate discussion on the use of eculizumab to potentially prevent serious complement-mediated vascular damage for chemotherapy-induced TMA in pediatric cases.

## Abbreviations

ADAMTS 13: A disintegrin and metalloproteinase with a thrombospondin type 1 motif, member 13
aHUS: Atypical hemolytic uremic syndrome
ARDS: Acute respiratory distress syndrome
CNS: Central nervous system
CSF: Cerebrospinal fluid
CT: Computed tomography
GiHUS: Gemcitabine-induced hemolytic uremic syndrome
HSCT-TMA: Hematopoietic stem cell transplantation-associated thrombotic microangiopathy
HUS: Hemolytic uremic syndrome
TMA: Thrombotic microangiopathy
